# Nanoparticles‐assisted delivery of antiviral‐siRNA as inhalable treatment for human respiratory viruses: A candidate approach against SARS‐COV‐2

**DOI:** 10.1002/nano.202000125

**Published:** 2020-10-12

**Authors:** Ata Ullah, Javaria Qazi, Lutfur Rahman, Antonios G. Kanaras, Waheed S. Khan, Irshad Hussain, Asma Rehman

**Affiliations:** ^1^ National Institute for Biotechnology and Genetic Engineering Faisalabad Pakistan; ^2^ Department of Biotechnology Quaid‐i‐Azam University Islamabad Pakistan; ^3^ Physics and Astronomy Institute for Life Sciences University of Southampton Southampton SO171BJ UK; ^4^ Department of Chemistry and Chemical Engineering SBA School of Science & Engineering (SBASSE) Lahore University of Management Sciences (LUMS) Lahore Pakistan

**Keywords:** Corona pandemic, human respiratory viruses, lipid nanoparticles, multifunctional nanocarriers, SARS‐CoV‐2, siRNA delivery

## Abstract

The current pandemic of coronavirus disease 2019 (COVID‐19) caused by severe acute respiratory syndrome coronavirus‐2 (SARS‐CoV‐2) has challenged healthcare structures across the globe. Although a few therapies are approved by FDA, the search for better treatment options is continuously on rise. Clinical management includes infection prevention and supportive care such as supplemental oxygen and mechanical ventilatory support. Given the urgent nature of the pandemic and the number of companies and researchers developing COVID‐19 related therapies, FDA has created an emergency program to move potential treatments with already approved drugs to patients as quickly as possible in parallel to the development of new drugs that must first pass the clinical trials. In this manuscript, we have reviewed the available literature on the use of sequence‐specific degradation of viral genome using short‐interfering RNA (siRNA) suggesting it as a possible treatment against SARS‐CoV‐2. Delivery of siRNA can be promoted by the use of FDA approved lipids, polymers or lipid‐polymer hybrids. These nanoparticulate systems can be engineered to exhibit increased targetability and formulated as inhalable aerosols.

## Graphical abstract

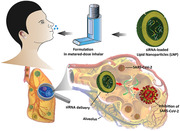

## 1 BACKGROUND

**FIGURE 1:**
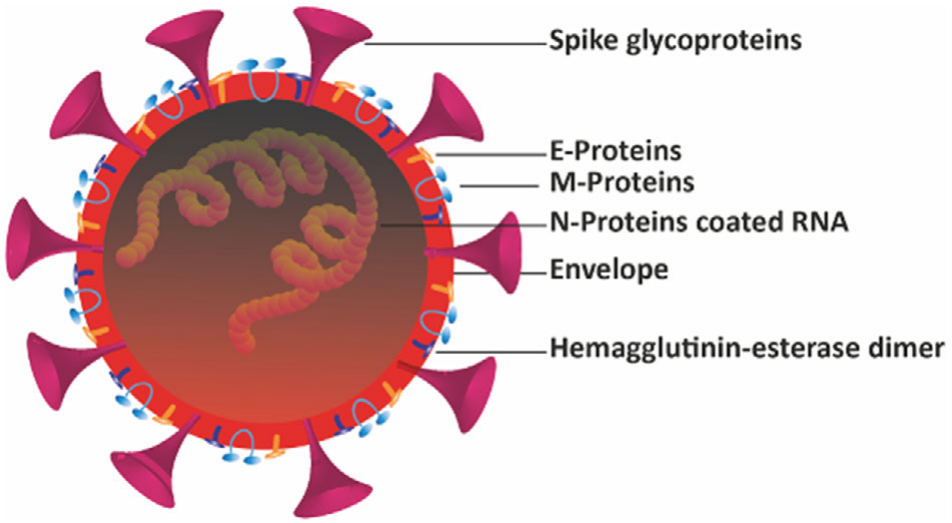
Structure illustration of SARS‐CoV‐2. This figure depicts the spherical morphology of the SARS‐CoV‐2 with three important components; Spikes, envelope and N‐protein coated nucleic acids. Other components includes: M/E proteins and Hemagglutinin‐esterase dimer

The outbreak of novel severe acute respiratory syndrome coronavirus‐2 (SARS‐CoV‐2) has challenged the global health structure and forced most of the people to quarantine to avoid rapid spreading. Human respiratory tract infections caused by viruses are commonly described as flu. Flu‐like symptoms or “Influenza‐like illness” (ILI) is a nonspecific respiratory illness characterized by fever, fatigue, cough, and other symptoms that stop within a few days, as the disease is usually self‐limiting. However, most of the cases of ILI are not caused by influenza viruses but by other viruses including rhinoviruses, human‐respiratory syncytial virus (RSV), adenoviruses, human parainfluenza viruses and corona viruses. Recently severe acute respiratory infection causing coronaviruses (SARS‐CoV), Middle East respiratory syndrome CoV (MERS‐CoV) and now SARS‐CoV‐2.^[^ ^1^ ^]^ Being a member of *coronaviridae* family, SARS‐CoV‐2 has enveloped spherical or pleomorphic shape with diameter ranging from 60‐140 nm and consists of four structural proteins, i.e., spike surface glycoproteins (S), envelope proteins (E), nucleocapsid proteins (N) and matrix proteins (M) (Figure 1).^[^ ^2^ ^]^ The genome of corona viruses (CoVs) has 6‐11 different open‐reading frames (ORFs). The first ORF is large enough to occupy two‐thirds of the viral RNA that encodes sixteen non‐structural proteins and two polyproteins such as pp1a and pp1ab, while the rest of ORFs are responsible for structural and accessory proteins. SARS‐CoV‐2 genome consists of non‐segmented, single‐stranded positive sense RNA having approximately 30,000 nucleotides.^[^ ^3^ ^]^ Various therapeutic approaches have been used for the treatment of ILI including antiviral drugs, monoclonal/polyclonal antibodies and RNA‐interference. FDA approved Oseltamivir in 1999 as a suitable antiviral agent for the treatment of ILI followed by ribavirin and aerosolized ribavirin. More recently, research has focused on the development of broad‐spectrum antiviral agents (BSAAs) to target multiple viruses by one drug. So far, there are reports of 120 BSAAs either under clinical trials or approved antiviral drugs.^[^ ^4^ ^]^ Among these BSAAs, Remdesivir and Chloroquine were reported as effective drugs against SARS‐CoV.^[^ ^5^ ^]^ In parallel, serological based therapeutics classified as convalescent‐plasma therapy and mono/polyclonal or chimeric monoclonal antibodies therapy is used against ILIs. The use of convalescent‐plasma therapy dates back to Spanish influenza pandemic (1918‐1920), and it has been reported as very promising against SARS‐CoV‐2 infection.^[^ ^6^ ^]^ In past few years, plethora of humanized and chimeric monoclonal antibodies have been discovered and used against respiratory syncytial virus (RSV), influenza virus, as well as SARS and MERS coronaviruses.^[^ ^7^ ^]^

Similarly, siRNA acts as broad‐spectrum antiviral therapeutics that can be engineered to target various viral genotypes and strains in different organs.^[^ ^8^ ^]^ siRNAs have exclusively been evaluated in vivo and in vitro in transfected cultured cells, mice and even non‐human primates for sequence‐specific inhibition of target viral RNA.^[^ ^9^ ^]^ This manuscript highlights the possibility of using siRNA as alternative approach to treat human respiratory virus infections as well.^[^ ^10^ ^]^ Particularly, ILI causing respiratory viruses are the ideal target for antiviral‐siRNA therapy approach because the upper respiratory tract and lungs are relatively easy to target.^[^ ^8c^ ^]^

## 2 CURRENT STATUS AND FUTURE DIRECTIONS OF THERAPEUTICS AGAINST COVID‐19

Till now, more than 2995 trails of SARS‐CoV‐2 have been posted in *ClinicalTrials.gov*, ranging from the use of previously existing antiviral drugs to novel diagnostics tools and bioimaging. Some antimalarial drugs such as chloroquine or hydroxychloroquine have also been used to battle against COVID‐19.^[^ ^11^ ^]^ However, they have not been very effective so far. Convalescent‐plasma therapy^[^ ^12^ ^]^ and antibodies^[^ ^6a^ ^]^ have also been reported for the treatment and Johns Hopkins University has received FDA approval to test plasma therapy. Tekda, a pharmaceutical company from Japan, has announced the use of anti‐SARS‐CoV‐2 polyclonal hyperimmune globulin (H‐IG) for the treatment of high‐risk individuals with COVID‐19. Furthermore, Regenen has pursued a strategy to develop monoclonal antibodies derived from genetically modified humanized mouse, which could be potentially used to treat COVID‐19. Wang et al. have reported human monoclonal antibodies that could bind to SARS‐CoV‐2 in order to inhibit their infection.^[^ ^13^ ^]^ Recently, US‐FDA approved the drug Remdesivir for the treatment of COVID‐19. Other supporting agents such as, azithromycin, ascorbic acid, and IL‐6 can also be used in combination to Remdesivir to enhance treatment efficacy.^[^ ^14^ ^]^

Coming towards the vaccine development, the US public health leader and director of National Institute of Allergy and Infectious Diseases (NIAID), Anthony Fauci has recently announced that “It will take at least 1–1.5 years to have a vaccine that we can use against novel coronavirus.” Recently, the news highlighted 6 vaccines i.e., Oxford vaccine candidate “ChAdOx1 nCoV‐19,” Massachusetts‐based Moderna vaccine, Beijing‐based Sinovac Biotech vaccine “PiCoVacc,” Pfizer and BioNTech vaccine “BNT162,” DNA‐based vaccine by Inovio Pharmaceuticals “INO‐4800,” and BCG vaccines, which are leading the global vaccine market for COVID‐19.^[^ ^10^, ^15^ ^]^

## 3 AN INSIGHT INTO THE USE OF RNA‐INTERFERENCE (RNAI) TO COMBAT HUMAN RESPIRATORY VIRUSES

RNA interference, mediated by artificially designed small interfering RNA (siRNA), is a potential strategy to inhibit RNA virus replication, expression of viral antigen and other genes.^[^ ^16^ ^]^ Antiviral siRNA therapy has many advantages over conventional anti‐viral drugs and vaccines, because; (i) it has rapid action with high efficiency and specificity at different stages of viral infection, (ii) only a sub‐stoichiometric amount of siRNA is enough to drastically decrease viral RNA load within 24 hours, (iii) specific length and high homology of siRNA with cognate viral RNA can selectively inhibit the particular gene. In 2001, human RSV was successfully targeted for the first time using siRNA.^[^ ^17^ ^]^ Similarly, intranasally administered siRNA was reported to reduce the viral titer of RSV and para‐influenza virus (PIV) up to 99%.^[^ ^18^ ^]^ Ge and co‐workers reported 20‐different siRNAs to inhibit all titer of human influenza virus in both Madin–Darby canine kidney (MDCK) cell lines as well as in chicken embryo.^[^ ^19^ ^]^ Importantly, the therapeutic potential of antiviral siRNA has also been evaluated against MERS‐CoV by designing two siRNAs (Smad7‐1 and Smad7‐2 siRNA) to knockdown MERS‐CoV and Smad‐7 gene in both human lung‐ and kidney‐cell lines. The Smad‐7 had effectively inhibited viral replication and virus induced cytopathic effects of host cells.^[^ ^20^ ^]^ Going a step beyond cell lines and using primates, i.e., *Rhesus macaques* as model, Li and colleagues reported the use of siRNA against SARS‐CoV. After the establishment of infection in monkey models, two siRNA duplex siSC2 and siSC5 targeting spike and non‐structural protein‐12 (ORF1b) coding regions of SARS‐CoV genome were intranasally administered resulting in reduced fever, diminished SARS‐CoV load and reduced acute diffuse alveoli damage.^[^ ^15b^ ^]^ Research progress in the use of anti‐SARS‐CoV siRNA therapy is indicated by the fact that at least three patents have been granted so far in this area,^[^ ^21^ ^]^ the details of which are provided in (Table 1). Sequence specific targeting of viral genome is the strength of antiviral siRNA therapy but cell specific targeted delivery of siRNA with subsequently inadequate endosomal escape is imperative to its success. Currently siRNA therapies are hindered by the ease of siRNA enzymatic degradation, fast clearance and inability to enter cells. Nevertheless, these issues can be significantly addressed using advanced nanocarriers including lipids, polymers or their hybrid nanoparticles, nanohydrogels, superparamagnetic iron‐oxide nanoparticles (SPIONs) and even functionalized gold nanoparticles.^[^ ^22^ ^]^

**TABLE 1:** Representative antiviral siRNA data from Chinese patent CN101173275, CN1648249, and US patent US20050004063

Patent	siRNA	Sequence	Target region/gene
CN101173275	siRNA‐M1	5′‐GGGUGACUGGCGGGAUUGCGAU‐3	460‐480 bp
	siRNA‐M2	5′‐GGGCGCUGUGACAUUAAGGAC‐3	
CN1648249	8*	5′‐CGUCGCAGCGUGUAGGCACUA‐3′	M protein gene
	51*	5′‐AACGGUUUACGUCUACUCGCA‐3′	N protein gene
	56*	5′‐AACGUACUGCCACAAAACAGC‐3′	E protein gene
US20050004063	SARSi‐1	5′‐GUGAACUCACUCGUGAGCUCTT‐3′	512‐531 bp of replicase A1 region
	SARSi‐2	5′‐GUACCCUCUUGAUUGCAUCTT‐3′	586‐604 bp of replicase A1 region
	SARSi‐3	5′‐GAGUCGAAGAGAGGUGUCUTT‐3′	916‐934 bp of replicase A1 region
	SARSi‐4	5′‐GCACUUGUCUACCUUGAUGTT‐3′	1194‐1213 of replicase A1 region
	SARSi‐5	5′‐CCUCCAGAUGAGGAAGAAGTT‐3′	3028‐3046 bp of replicase A region
	SARSi‐6	5′‐GGUGUUUCCAUUCCAUGUGTT‐3′	5024‐5042 bp of replicase A region

## 4 RECENT PROGRESS IN NANOMATERIALS ENGINEERING FOR EFFECTIVE SIRNA DELIVERY

Being negatively charged and unstable, the in vivo delivery of siRNA is still a major obstacle in therapeutic applications. For example, naked siRNA is highly sensitive to ubiquitous nucleases and can be degraded before reaching to the target and hence may not be much efficient for targeted RNA inhibition.^[^ ^23^ ^]^ In some cases, the deadliest pathogenic viruses (i.e., adenoviruses) have been used as a siRNA carrier to ensure siRNA protection and the retention of their biological activity. But a major problem associated with viral based delivery has been immuno‐response against the administered virus and, in some viral vector‐like lentivirus, random insertion in chromosomes leading to gene dysfunctions. Therefore, efficient, non‐toxic, biocompatible and targeted delivery methods remain an urgent goal.^[^ ^24^ ^]^ Opportunities to develop such methods have been made possible due to a number of potential strategies offered by nanotechnology. The use of various types of organic nanoparticles as carriers for the delivery of therapeutic payload offers remarkable advantages due to their excellent in vivo retention by reducing enzymatic degradation and sequestration by reticulo‐endothelial systems. Earlier, the designed nanocarriers were used for the systemic delivery of siRNA but now‐a‐days the focus is the development of multifunctional nanocarriers (lipid/polymers or their hybrid nanoparticles, etc.) for localized delivery of siRNA to reduce the systematic toxicity of siRNA.^[^ ^25^ ^]^

Appropriately functionalised nanoparticles provide immunochemistry inert‐surfaces contact with the biological system in addition to possessing the high permeability and retention efficiency to enhance their efficacy and deposition in targeted tissues.^[^ ^26^ ^]^ In this regard, a plethora of organic, inorganic and metal nanoparticles have been reported for targeted in‐vivo delivery of therapeutics biomolecules such as silica, gold, dendrimers, polymeric and lipid nanoparticles, etc.^[^ ^27^ ^]^ However, among these, lipids and polymers are considered as promising materials for designing of multifunctional nanocarriers for siRNA delivery because of their better biocompatibility and biodegradable nature. For instance poly(glycolic acid) (PGA), poly(lactic acid) (PLA), polycaprolactone (PCL) and their copolymers PLGA have been approved by FDA and most widely used for in vivo siRNA delivery.^[^ ^27^, ^28^ ^]^ Similarly, lipid nanoparticles have been recognized as the most proficient siRNA delivery system. Various types of lipid nanocarriers have been used for delivery of siRNA including solid‐lipid nanoparticles, nanostructured‐lipids and liposomes, etc.^[^ ^29^ ^]^ Gomes‐da‐Silva and co‐workers have reported that lipid nanocarriers protect siRNA from serum nucleases, prolong their circulation and enhance their access to target sites.^[^ ^30^ ^]^ The aerosol based delivery of plasmid DNA using cationic liposomes was first time reported in 1992,^[^ ^31^ ^]^ while the same method has been used for the delivery of siRNA by Thomas and co‐workers.^[^ ^32^ ^]^ Moreover, it has been well documented that polycationic‐lipids or polymers maintain low endosomal pH through an increase in influx of protons and water and thus, lead to the rupturing of endosome to drop therapeutic payload into cytosol.^[^ ^33^ ^]^ Furthermore, the antiviral‐siRNA delivery through commercially available cationic lipid formulations such as lipofectin, oligofectamine, lipofectamine (Invitrogen), RNAifect (Qiagen), and TransIT TKO (Mirus) have also been reported.^[^ ^33^, ^34^ ^]^ PLGA nanoparticles have been found to be suitable for inhalable freeze‐dried powder and liposomes for aerosol based pulmonary delivery of antiviral‐siRNA.^[^ ^35^ ^]^

A lipid based multifunctional envelope‐type nanodevice (MEND) has been utilized for delivery of siRNA into lungs epithelium to treat lungs metastasis. The MEND is guided to lungs by surface modification with GALA‐peptide that binds with the sialic acid‐terminated sugar chains on the lung endothelium.^[^ ^36^ ^]^ Recently, a lipidoid‐polymer hybrid nanocarrier has also been reported for pulmonary delivery of siRNA.^[^ ^37^ ^]^ Other inhalable lipidoid‐polymer hybrid nanocarriers have also be employed for siRNA delivery. For example, a spray dried siRNA‐loaded lipidoid‐PLGA hybrid nanocarrier as self‐inhalable formulation, stabilized with mannitol, has been reported.^[^ ^38^ ^]^ Furthermore, cholestrol‐conjugated lipid nanoparticles (LNPs) have been recently reported by Eygeris et al. as potential nanocarriers for the delivery of an mRNA vaccine against SARS‐CoV‐2. Many of LNPs based mRNA based vaccines are currently in clinical trials as detailed in Table 2.^[^ ^39^ ^]^ Interestingly, histidine‐lysine co‐polymer (HKP) and spermine‐liposome conjugates (SLiC) based nanocarriers have also been documented in a US patent 2019/0030187A1 for the delivery of siRNA to target specific genes in SARS‐CoV genome. After the onset of clinical signs in an infected mice and non‐human primates (i.e., *Rhesus macaques* as model organisms), they were treated with intranasally administered nano‐formulated antiviral siRNA with very promising results.^[^ ^40^ ^]^

**TABLE 2:** Lipid nanoparticles as vector for delivery of mRNA vaccine for the treatment of COVID‐19

Candidate vaccine	Developer	Target	Status
3 LNP‐mRNAs	BioNTech/Fosun Pharma/Pfizer	SARS‐CoV‐2	Phase ½ 2020‐001038‐36
LNP‐encapsulated mRNA	Moderna/NIAID	SARS‐CoV‐2	Phase 1/2 NCT04283461
LNP‐encapsulated mRNA vaccine encoding *S*‐protein	Moderna	SARS‐CoV‐2	Phase 1 NCT04283461
LNP‐encapsulated mRNA cocktail encoding VLP	Fudan university/Shanghai Jointing, University/RNAcare, Biopharma	SARS‐CoV‐2	Pre‐clinical
LNP‐encapsulated mRNA encoding RBD	Fudan university/Shanghai JioTong, University/RNAcare, Biopharma	SARS‐CoV‐2	Pre‐clinical
LNP‐encapsulated mRNA	University of Tokyo/Daiichi‐Sankyo	SARS‐CoV‐2	Pre‐clinical
Liposome‐encapsulated mRNA	BIOCAD	SARS‐CoV‐2	Pre‐clinical
LNP‐mRNA	Translate Bio/Sanofi Pasteur	SARS‐CoV‐2	Pre‐clinical

## 5 FUTURE PROSPECTS FOR THE USE OF ENGINEERED NANOMATERIALS FOR ANTIVIRAL‐SIRNA DELIVERY AGAINST SARS‐COV‐2

Above mentioned findings suggest the possibility to treat COVID‐19 infection through self‐inhalable nanoencapsulation of antiviral‐siRNA. Antiviral‐siRNA therapies have been successfully applied against various deadly pathogenic human respiratory viruses including SARS‐CoV, MERS‐CoV, RSV and influenza virus using mice and non‐human primates as model organisms.^[^ ^15^, ^18^, ^20^, ^41^ ^]^ The use of siRNA therapeutics against SARS coronavirus is already well established and patented,^[^ ^21b^ ^]^ and this article focusses on the rationale design of nanocarriers for siRNA therapeutics that could be safely and efficiently applied for the treatment of human respiratory viral infections. The use of nanocarriers has great potential to adequately address the current challenges of stability and fragile nature of siRNA. These nanocarriers may ultimately lead to the development of efficient delivery systems that can intelligently deliver the intact siRNA payload into targeted tissue or an organ in the body.

**FIGURE 2:**
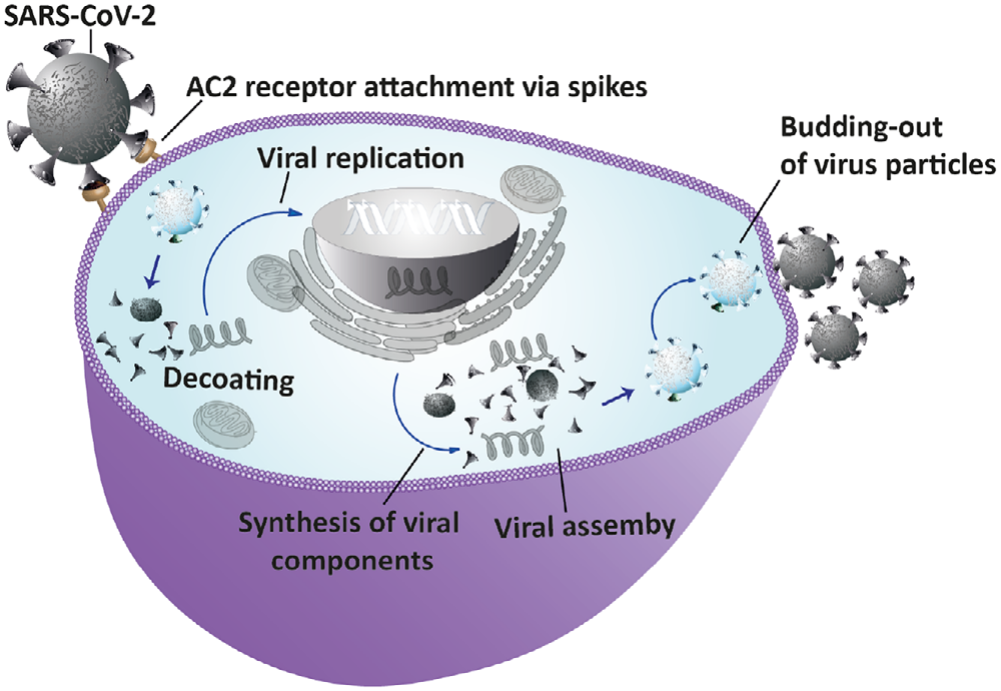
Figure represents the replication of SARS‐CoV‐2 in host cell. After receptor‐mediated invasion into cell the virus disintegrates and releases their RNA into cytoplasm for replication. Then, the viral RNA enters in the cell nucleus and hijacks the cellular machinery for its replication. After replication of viral genome and their associated components, new virus particles are formed, which are exocytosed from cells

The virologic study shows that SARS‐CoV‐2 is primarily transmitted from infected people to the healthy ones by close contact, through respiratory droplets or by contact with contaminated surfaces and objects. In upper respiratory tract, the virus gets ACE2‐recepeter mediated cellular entry and starts its replication (Figure 2).^[^ ^42^ ^]^ Roughly, SARS‐CoV‐2 infection can be divided into 3‐stages such as: stage‐I is an incubation period and generally asymptomatic with or without detectable virus. The patient with stage‐I is known as stealth carrier and can unknowingly spread the virus. The incubation time of the virus is on average 5‐6 days but it can be up to 14 days.^[^ ^43^ ^]^ Stage‐II is mild symptomatic period with the presence of virus,^[^ ^44^ ^]^ and the clinical data reveals that the biological samples from infected patients show the highest viral load in upper respiratory tract such as nose and throat.^[^ ^45^ ^]^ The stage‐III results in severe respiratory symptoms with higher viral load that, depending on the patients’ immune system, may result in organs failure and thus, the death of patients.^[^ ^44^ ^]^

A plausible treatment of SARS‐CoV‐2 infection could be the use of cationic‐liposomal encapsulation of antiviral‐siRNA and their aerosol formulation for intranasal or intratracheal administration through metered‐dose inhaler. The antiviral siRNA can be effectively designed for target hybridization to the accessible sites within the viral RNA hence avoiding off‐target effects. A few excellent computer algorithms and empirical testing based combinatorial methods are also available to define potent antiviral‐siRNAs.^[^ ^46^ ^]^

After intelligent designing of siRNA, their encapsulation in cationic lipid nanocarriers can be customized into site‐specific delivery for a localized effect as discussed above. Therefore, the lipid/polymer based nanocarriers’ surface can be modified with functional molecules (i.e., antibodies or aptamers) that could target cell or tissue specific surface markers. For example, the lung cells have alveoli‐specific surface markers such as alveolar epithelial type‐I and II cell‐specific markers (AT‐I and AT‐II). AT‐I cells are found in abundance on the internal surface of lungs and cover ∼95% surface area of the lung, the rest of 2–5% are covered by AT‐II.^[^ ^47^ ^]^ Thus, antibodies against these two receptors could be conjugated to the surface of nanocarriers to direct their binding to the alveoli cells specific surface markers and subsequently deliver the siRNA payload into their cytoplasm. The targeted delivery may also be achieved through targeting of the ACE2 surface marker, the main entry point of COVID‐19.^[^ ^44^, ^48^ ^]^ Interestingly these receptors are present in abundance in human lower respiratory tract, lungs, venous endothelial cells, arterial, arterial smooth muscle cells, brain, kidney, stomach, small intestine, and other tissues and organs. The clinical data shows that these tissues and organs are at high risk to be infected and are generally prone to damage during COIVD‐19 infection.^[^ ^49^ ^]^ Thus, lipids or lipid/polymer hybrid nanocarriers functionalized with anti‐ACE2 antibodies could be utilized to target the ACE2 cell surface markers and deliver siRNA cargos. Another advantage of the conjugation of targeting moiety (antibodies) against ACE2 markers to nanocarriers could be to target all the cells and organs having ACE2 surface markers and, therefore, broadens the scope of siRNA based therapy spectrum as compared to anti‐ AT‐I and AT‐II antibodies functionalized nanocarriers that would only target lung cells.

**FIGURE 3:**
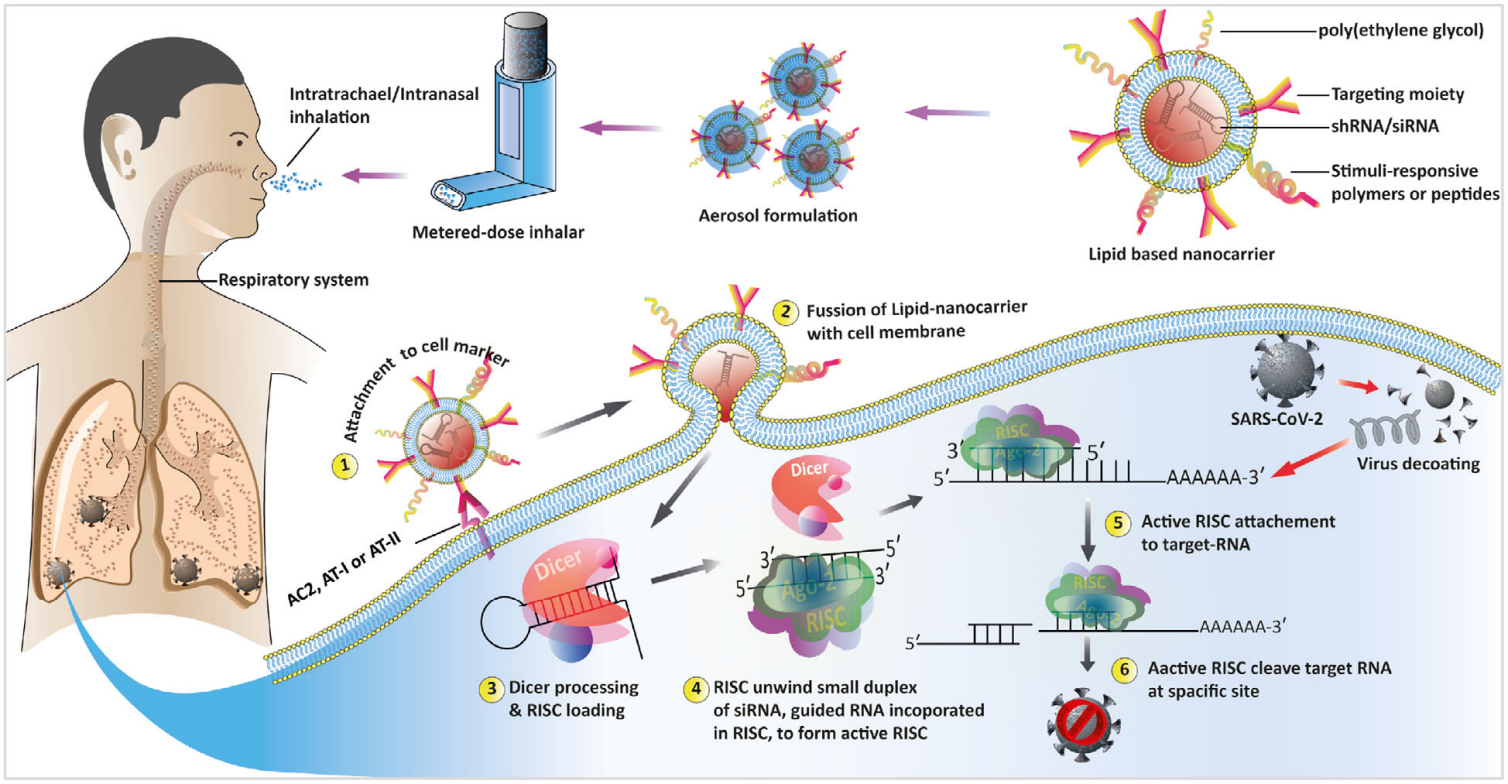
A schematic illustration of the proposed treatment of SARS‐CoV‐2 through the use of multifunctional nanocarriers that deliver antiviral siRNA into the respiratory system to combat viral infection. The first step is the encapsulation of siRNA in nanocarriers, its formulation in aerosols and their packing like in, metered‐dose inhaler to facilitate their pulmonary delivery. Surface of multifunctional lipid based nanocarrier modified with targeting moiety to AT‐I, II or those have ACE2 receptor, customize their delivery into lungs cells. The second step is the attachment of the siRNA‐encapsulated lipid‐nanocarrier to desired cells/tissues. In the cytoplasm the exogenously delivered siRNA activates the RNA‐interference pathway which chop‐down targeted sites in viral genome leads to inhibit their infection

Moreover, the surface of nanocarriers could be modified with poly(ethylene glycol) that has been shown to stabilize nanoparticles and prolong their circulation time in vivo.^[^ ^34a^ ^]^ Stimuli responsive nanocarriers have also been well documented for siRNA delivery. For instance, the conjugation of pH‐sensitive histidine‐lysine peptide to nano‐delivery systems has been shown to assist the endosomal release of siRNA into cytosol to induce RNA interference pathway.^[^ ^50^ ^]^ Hence such types of stimuli responsive features can be incorporated to the nanocarriers for efficient release of siRNA payload on targeted site. Once the RNA interference pathway becomes active, it leads to the cleavage of viral RNA at the targeted sites to block the viral replication that reducing the viral load and helping to cure the viral infection (Figure 3).

## 6 CONCLUSION AND FUTURE OUTLOOK

COVID‐19 pandemic continues to unfold and urges multiple biotechnology companies, research institutions and the governments to consolidate their efforts on the development of efficacious therapeutic approaches against SARS‐CoV‐2. In response to the current health emergency, US FDA and CDC offered extraordinary flexibility to the companies and research labs to develop potent antiviral/drug/vaccines against COVID‐19. Healthcare companies across the globe are working hard to develop the possible treatment for COVID‐19 with some candidate drugs on the fast track expediting the clinical trials. In this context, small interfering RNA is considered as a potent antiviral‐therapeutic agent due to sequence‐specific degradation of target RNA (viral) with high specificity and efficacy. Various antiviral‐siRNA treatments have been reported against deadly pathogenic human respiratory viruses (SARS‐virus, Influenza virus) by targeting their virulence genes in their genome such as papain‐like protease (PL_pro_), RdRP, spike proteins, structural genes (M and N) and non‐structural genes (nsp) such as nsp‐2, nsp‐10, and nsp‐15. The siRNA based silencing and knockdown of these viral genes have potentially been studied using mice and non‐human primates as model organisms and the majority of them are currently in human clinical trials. Reviewing the relevant literature for the siRNA‐based treatment of human respiratory viruses, we are suggesting the following plausible options:

i. Sequence specific siRNA is proven to be effective against human respiratory viruses and hence can be potentially utilized against SARS‐CoV‐2.
ii. siRNA encapsulation in multifunctional nanocarriers is very likely to address the siRNA delivery issues and facilitate their controlled, sustained and unprecedented stimuli responsive targeted delivery.
iii. Finally the aerosol formulation for intranasal or intratracheal administration of engineered siRNA nanocarriers through metered‐dose inhaler is suggested as an effective route of administration to treat SARS‐CoV‐2.
